# Impact of eating disorders on paid or unpaid work participation and performance: a systematic review and meta-analysis protocol

**DOI:** 10.1186/s40337-021-00525-2

**Published:** 2022-01-15

**Authors:** Fatima Safi, Anna M. Aniserowicz, Heather Colquhoun, Jill Stier, Behdin Nowrouzi-Kia

**Affiliations:** Department of Occupational Science and Occupational Therapy, Temerty Faculty of Medicine, University of Toronto, 160-500 University Avenue, Toronto, ON M5G 1V7 Canada

**Keywords:** Eating Disorders, Work, Work Participation, Work Performance, Occupational Therapy, Anorexia Nervosa, Bulimia Nervosa, Binge Eating Disorder

## Abstract

**Background:**

Eating disorders (ED) can reduce quality of life by limiting participation and performance in social and occupational roles, including paid or unpaid work. The association between ED pathologies and work participation and performance must be well understood to strengthen vocational rehabilitation programmes and prevent occupational disruptions in the ED population. The aims of this study are: (1) to examine the degree of association between ED pathologies and work participation and performance in 15-year-olds and older; (2) to highlight the specific ED symptoms that are most correlated with changes in work performance and participation; (3) to compile the most common metrics and assessments used to measure work participation and performance with ED.

**Methods:**

Medline, Embase, CINAHL, Web of Science, PsycINFO, and Cochrane Library will be searched for observational and experimental studies that meet the following criteria: (1) a clinical sample of typical or atypical ED; (2) paid or unpaid employment or training; (3) an association between ED pathologies and work participation or performance. Unpublished data will also be examined. Title and abstract, and full-text screening will be conducted in duplicate. Risk of bias and quality of evidence assessments will be completed. A random-effect meta-analysis will be performed.

**Discussion:**

This synthesis can clarify knowledge and gaps around the impact of ED on work functioning, thereby allowing better evaluation, improvements and development of current workplace assessments, interventions, and policies.

***Trial registration*:**

The registration number for this systematic review on PROSPERO is CRD42021255055.

## Background

Eating disorders (ED) are among the most persistent and disabling mental health disorders worldwide [1–7]. The Diagnostic and Statistical Manual of Mental Disorders 5th Edition (DSM-5) and the International Classification of Diseases Revision 11 (ICD-11) both highlight an association between ED and dysfunctions in everyday occupations and social and professional roles [3, 8–14]. Many studies have also highlighted physical, psychological, and social dysfunctions related to ED [3, 4, 10, 12, 13, 15–17]. In addition, ED have a substantial financial and economic burden on a global scale [4, 16].

Recent studies related to the financial burden of ED showed that their effect on the economy is profound due to loss of productivity in the workplace [4, 16]. Loss of productivity was defined in those studies as reduced task completion, attendance, and fulfillment of job demands by individuals in their workplace [4, 16]. Streatfield et al. [16] found that during the fiscal year of 2018 to 2019 in the United States, the total tangible economic cost of ED was around 64.7 billion USD, with 48.6 billion USD due to loss of individual productivity and the remaining 6.7 billion USD attributed to loss of caregiver productivity. A similar study in South Korea by Lee et al. [4] found that in a period of six years, the total economic cost of ED was around 5.5 billion USD. Loss of productivity due to work absence comprised part of that value.

Similarly, studies examining employment patterns in individuals with ED highlight a negative impact on work participation and performance, from decreasing productivity to contributing to unemployment. A longitudinal study found that 1 in 4 participants with ED reported unemployment due to a psychiatric disability 18 years after diagnosis in adolescence [18]. Meanwhile, those who were employed relied on sick pensions and extended sick leaves as a result of their ED [18]. Other studies on individuals with anorexia nervosa (AN) reported over 20% of females and 10% of males relying on sick pensions, sick leave, and social welfare for at least six months post inpatient ED treatment [7, 19]. The National Eating Disorder Association (NEDA) also highlighted the impact of ED in the workplace, stating that ED can lead to declines in productivity in both diagnosed and undiagnosed workers due to increased emotional and mental demands [20]. Finally, social disability and unemployment levels in individuals with ED have been reported to be as high as those of individuals with other severely disabling and chronic mental health disorder such as schizophrenia and personality disorders [12, 21–23].

Despite numerous reports on the negative impact of ED on employment, the association between work and ED is not yet well understood. Specifically, the strength of the association between ED and work participation and performance, the reasons behind the negative effects, and the methodologies used to measure those effects require further investigation. To date, no knowledge syntheses have examined the association between ED and work participation and performance. As a result, no conclusive descriptive or statistical data are available about this relationship. Also, no knowledge syntheses have examined how researchers measure and define concepts related to functioning at the workplace. The abovementioned gaps may be limiting the suitability of current intervention programmes and workplace accommodation policies for individuals with ED. Therefore, the association between ED pathologies and work participation and performance must be well understood to strengthen vocational rehabilitation programmes, prevent occupational disruptions, and appropriately support individuals with ED.

The proposed systematic review and meta-analysis aims to broaden and clarify the knowledge base around the effects of ED on work participation and performance by seeking answers to the following research questions: (1) What is the degree of association between ED pathologies and work participation and performance in individuals aged 15 years and older? (2) Which specific ED symptoms are most correlated with changes in work participation and performance? (3) What are the metrics and assessments used by clinicians to measure work participation and performance in the context of ED? The third research question is included for the purpose of completing the meta-analysis, as a specific statistical technique cannot be determined without appreciating the types of metrics and data available about this topic first. This protocol is developed to ensure rigour, comprehensiveness, and transparency when conducting the proposed systematic review and meta-analysis [24].

## Methods

### Study design and registration

A systematic review and meta-analysis will be conducted to synthesize all empirical evidence on the relationship between ED and work participation and performance. The registration number for this systematic review is CRD42021255055 on PROSPERO. At the time of registration, no knowledge syntheses of similar aims were found. The Cochrane Handbook for Systematic Reviews of Interventions and the Preferred Reporting Items for Systematic Reviews and Meta-analyses Protocols (PRISMA-P) checklist were followed to prepare this protocol [25, 26]. Reporting in the actual systematic review and meta-analysis will also follow The Preferred Reporting Items for Systematic Reviews and Meta-analyses (PRISMA) guidelines [27].

### Search strategy

This systematic review will search the following six databases for existing articles about ED and work participation and performance: Medline (Ovid), Embase (Ovid), CINAHL (Ebsco), Web of Science, APA PsycINFO (Ovid), and Cochrane Library (Wiley). These databases were chosen because they allow for advanced searching in healthcare and mental health literature. Unpublished data, which encompasses conference proceedings, dissertations, unpublished complete trials, research reports, and any specific outcomes not included in published trials, will be sought as well [28]. Backward and forward citation searching will be performed. Backward citation tracking will be done by hand-searching the reference list of included studies. Forward citation searching will be done using the Web of Science Core Collection citation index to examine all subsequent articles that cited the included studies. The free reference management tool, Zotero, will be used to store and organize the references [29].

The search syntax was developed by the first author and peer-reviewed by the team and a health science librarian. The Canadian Agency for Drugs and Technologies in Health Peer Review Checklist for Search Strategies was used to guide the peer review process [30]. Appendix 1 outlines the search concepts and syntax for each database. No restriction to the year of publication and language will be placed.

### Study eligibility criteria

#### Inclusion criteria

All studies must examine a clinical sample of typical or atypical ED as per the diagnostic criteria of ‘Feeding and Eating Disorders’ in the DSM-IV, DSM-5, ICD-10 or ICD-11 (e.g. AN). The target population for this study is male or female 15-year-olds and older. The target population starts from 15 for two reasons. First, 15 is the minimum age requirement for legal employment according to the Minimum Age Convention (No. 138) established by the International Labour Organization [31]. Second, ED symptoms typically emerge in mid-adolescence, warranting the inclusion of working teens in the exploration process.

The participants in the primary articles must be completing paid or unpaid work or training as part of a job or a volunteering position. In the literature, work often encompasses finding and/or keeping a paid or unpaid job as well as completing training in preparation for employment [32]. Both work participation and work performance are generally examined when work is conceptualized as an activity that requires mental or physical effort to achieve a purpose [33]. The definitions, and even the terminology used to refer to work participation and work performance, differ among studies [34–40]. For the purpose of the proposed systematic review, the definitions outlined by Lagerveld et al. [39] will be used. Work participation will refer to a person’s tendency to be present in the workplace to fulfill roles and responsibilities of paid or unpaid jobs. On the other hand, work performance, dubbed ‘work functioning’ in Lagerveld et al.’s [39] study, will refer to a person’s ability to carry-out or complete required tasks of paid or unpaid jobs either fully or partially.

Included studies must report an association between ED symptoms and work participation or performance as per the abovementioned definitions using either between or within group comparisons. The assessments or metrics used to measure work participation or performance must be clearly described because those will help determine the statistical techniques pursued in the proposed meta-analysis. Indicators of work participation and performance can span, but are not limited to, productivity, absenteeism, presenteeism, workload completion, and the quality of work.

#### Exclusion criteria

Studies will be excluded if their population has a dual diagnosis of neurodevelopmental conditions (e.g. autism) or conditions marked by severe physical changes (e.g. cancer, female triad syndrome, stroke). The above mentioned criteria are in place to reduce the interference of third variables on the outcomes. However, studies where participants have a dual diagnosis of other mental health disorders (e.g. substance use, anxiety, depressive, bipolar, personality) will not be excluded given that ED are highly comorbid [1, 3, 4, 15, 16]. Thus, paucity of samples with a sole diagnosis of ED is highly likely. Also, studies with the following characteristics will be excluded: a population younger than 15 years of age is examined without splitting the reported outcomes between age groups; work is defined in terms of satisfaction or engagement without participation or performance markers; work is included as part of leisure, school, or household management activities; treatment interventions are the primary focus of the study. Finally, reviews, book chapters, and knowledge syntheses will be excluded, but their reference lists will be examined for primary articles that fit the eligibility criteria. Primary articles that are not peer-reviewed, and articles that do not offer English translations or full-text even after authors are contacted, will be excluded as well.

### Data collection

The systematic review will be carried out by a team consisting of registered occupational therapists (OTs), OT students, and methodological experts. Input from the advisory group will occur throughout the review process to ensure adherence to best practice and guidelines. Two independent reviewers will conduct screening and selection of articles against the eligibility criteria at two levels: (1) title and abstract screening, and (2) full text screening [41]. The two reviewers will meet at the beginning, midpoint, and final stages of the title and abstract screening to address uncertainties and revise the eligibility criteria based on retrieved abstracts if need be. This step is included to limit ambiguity in the selection process. Disagreements and discrepancies around article selection at both levels will be resolved through discussion between the two reviewers [41]. If consensus is not achieved between the reviewers, consultation with other team members will be arranged [41]. All non-English articles that appear from the search will be grouped under ‘Studies Awaiting Classification’ in the PRISMA flow diagram to inform readers of the availability of other potentially relevant reports [42, 43]. Similarly, reasons for exclusions will be reported in a table titled ‘Characteristics of Excluded Studies’ as per Cochrane’s recommendations [42]. The review management software, Covidence, will be used to maintain the quality assurance and organization of the data screening, collection, and analysis processes [24, 44].

### Data extraction

Data extraction will be conducted by two reviewers independently. Inter-rater reliability between the reviewers will be retrieved. The process will involve both reviewers extracting information from the first five studies that meet the inclusion and exclusion criteria using a data-charting form developed and validated by the team [41]. Then, the degree of agreement between the data extractors will be calculated using Kappa statistics [41]. The data-charting form will include: (1) author name(s); (2) publication year; (3) country of origin; (4) study design; (5) a description of the population (e.g. sample size, the ED diagnosis); (6) a description of the ED symptom(s); (7) a description of the work participation or performance outcome(s) (e.g. definition, assessment or metric used); and (8) the overall findings of the article (e.g. the association present, means, standard deviations, number of events and non-events). In the case of unclear or missing data, study authors will be contacted to provide clarifications. All disagreements and discrepancies will be resolved through discussions between the two reviewers, and if need be, by a third reviewer from the team [41].

### Risk of bias assessment

A risk of bias assessment for each included study will be done by two independent reviewers. For randomized control trials, Cochrane’s Risk of Bias 2 (RoB 2) Excel tool will be used [45, 46]. For cohort and case-control studies, the Newcastle Ottawa Scale will be used [47]. For cross-sectional studies and case series, the Joanna Briggs Institute (JBI) checklist will be used [48]. All included studies will be classified into high, moderate, or low risk of bias. Discrepancies will be addressed first through discussion between reviewers, and if necessary, through consultation with the rest of the team. Consensus data will be recorded on a separate form from the original to maintain transparency [41]. A priori for distinguishing between high, moderate, and low risk of bias using each critical appraisal tool will be followed. See Appendix 2 for priori scores adapted from the literature [46, 49, 50].

### Data synthesis

A descriptive numerical summary will be reported in a table titled ‘Characteristics of Included Studies’, illustrating the following parameters: (1) overall number of studies; (2) study author(s); (3) country of origin; (4) year of publication; (5) study design; (6) characteristics of participants, such as diagnosis and age; (7) measure(s) used to determine ED symptoms and their severity; (8) measure(s) or metrics used to determine the work participation and performance of participants; and (9) risk of bias category [41]. When reporting measures for work participation and performance, the specific indicators will be included (e.g. absenteeism, productivity) as well as the type of measures that yielded them (e.g. self-reports, performance-based observations) as per Cochrane recommendations [51]. A narrative synthesis will be completed to summarize the results following the Guidance on the Conduct of Narrative Synthesis in Systematic Reviews developed by Popay et al. [52]. Finally, the Grading of Recommendations, Assessment, Development and Evaluations (GRADE) framework will be used to report the strength of evidence for the association between ED and work participation and performance [53].

#### Meta-analysis

A random-effect meta-analysis will be performed if it can accommodate for the anticipated heterogeneity in the extracted data [54]. Also, a meta-regression will be performed to account for any clinical heterogeneity found due to the inclusion of comorbid disorders in the participant sample. Specifically, percent comorbid diagnosis will be set as a moderator when conducting the meta-regression. Heterogeneity will be measured using Cochrane’s Q and I^2^ statistics, where I^2^ of 0 to 40% will indicate no heterogeneity and I^2^ of 40% or above will indicate moderate to high heterogeneity [55]. The specific metrics that will be used to calculate independent effect sizes for work participation and performance will be determined based on the types of data available after extraction. Forest plots will be constructed to check for publication bias [56]. A sensitivity analysis will be conducted as well to determine the robustness of the findings, and will be presented using a summary table [57]. All statistical computations will be carried out using the free software R [58].

## Dissemination

The completed systematic review and meta-analysis will be presented at the World Federation of Occupational Therapists (WFOT) 2022 conference. The final manuscript of the systematic review will be submitted for publication in peer-reviewed journals.

## Discussion

The proposed review and meta-analysis will enhance the knowledge base around the impact of ED on work participation and performance by examining working teens and adults. Clarifications will be provided on what aspects of the disorder correlate with dysfunctions in the workplace and how researchers assess the extent of work participation and performance within the ED population. This knowledge synthesis will aim to present the conclusions through descriptive and statistical means to better summarize the impact of ED on work participation and performance.

Much is still unclear regarding how and why ED impair work participation and performance. Currently, it is sometimes stated that ED may be impairing workplace functioning because of low body weight. Low body weight often involves nutritional deficits and can limit attention, memory, and energy levels, which can then make work performance and participation difficult [10, 11, 59–64]. However, it is unlikely that low body weight is the only or even the main reason for the negative impacts of ED on work functioning. Poor work participation and performance have been observed in individuals who have achieved ‘recovery’ from an ED by maintaining a healthy body weight and abandoning abnormal eating behaviours [18, 65]. Similarly, evidence for poor work participation and performance exist for people with ED diagnoses not characterized by significant low body weight, such as bulimia nervosa (BN) [3]. Moreover, other mental health disorders not characterized by abnormal eating behaviours or weight have been shown to impair functioning in the workplace, such as depressive disorders, bipolar disorders and anxiety disorders [34, 36, 37, 40].

Since ED are highly comorbid with depression and anxiety, the impact of ED on work participation and performance may be partly due to accompanying mood disorders. Alternatively, the negative impacts of ED on work may be due to the psychological imbalances of ED rather than the physical deficits that are related to malnutrition and severe low body weight. In 2011, a meta-analysis by Ford et al. [35] found that the association between work performance and psychological health is moderate to strong, but the association between work performance and physical health is weak to moderate. Also, the meta-analysis suggested that the effects of dieting on work performance is weak. Since ED are marked by both physical and psychological dysfunctions, their impact on functioning in the workplace may be layered and distinct from other mental health disorders. Thus, comprehensively understanding the relationship between ED and work will be an important step in gaining a better appreciation of how mental and physical health may be impacting functioning in common everyday activities.

This systematic review may be of special interest to the fields of nursing, psychiatry, psychology, social work, and occupational therapy as each discipline plays a significant role in facilitating the journey of recovery in individuals with ED [66]. Since most patients with ED fall under the working-age group, participating in and preforming paid or unpaid work will likely be a relevant activity and a determinant of self-identity for that population. A healthy sense of identity and self-esteem has been described as an imperative component of facilitating recovery from ED [10]. This is because patients with high self-esteem and a strong sense of identity may experience less drive to hold on to their maladaptive ED labels and thus be more inclined to seek and cooperate with treatment [10, 67, 68]. Healthcare practitioners must be prepared to assess and address issues of work participation and performance optimally in the ED population should they come forward during therapeutic interactions [66].

A better understanding of the relationship between ED and work participation and performance may enable better evaluation, improvement and development of interventions and policies that target return to work, continuation of employment, and even prevention of dysfunctions in the workplace. Furthermore, having a better understanding of the types of dysfunctions experienced by individuals with ED may help identify new or more accurate pathways to recovery. It is important for individuals with ED to recover and be able to partake in their desired work instead of merely experiencing absence of symptoms, which does not always equate to resumption of satisfactory levels of function [6, 10, 65]. Finally, the findings of this proposed review may aid the development of future assessments that can predict and evaluate work participation and performance in individuals with ED.

Although this protocol outlines many strengths in the proposed systematic review methodology, such as the incorporation of validated risk of bias assessments for each type of study design, a peer-reviewed search strategy, and detailed inclusion and exclusion criteria, it contains some limitations that may threaten the comprehensiveness of this review. First, relevant evidence may be discounted when non-English articles are excluded from data extraction. Second, as heterogeneity in study designs, measurement tools, participant characteristics, and definitions of work participation and performance as study variables are anticipated, there may be limited studies from which conclusions and a quantitative meta-analysis can be drawn. Nevertheless, the completion of a systematic review around ED and work functioning is imperative to help address the needs of this persisting and growing population.

## Data Availability

Not applicable.

## Appendix 1

See Table 1.

**Table 1:** Search syntax for each database

*Medline (Ovid)*
1. work/ or work performance/ 2. return to work/ 3. employment/ or employment, supported/ or teleworking/ or occupations/ 4. absenteeism/ or efficiency/ or presenteeism/ or burnout, professional/ or workload/ 5. work capacity evaluation/ or employee performance appraisal/ 6. (work* or job* or employ* or career* or occupation* or vocation* or profession* or telework* or telecommut*).kf,tw 7. (absenteeism or presenteeism or (sick* adj2 presen*) or burnout).kf,tw 8. ((capacity or disability or function*) adj2 (evaluation or assessment or scale)).ab,kw,ti 9. "feeding and eating disorders"/ or anorexia nervosa/ or avoidant restrictive food intake disorder/ or binge-eating disorder/ or bulimia nervosa/ or diabulimia/ or night eating syndrome/ or pica/ or rumination syndrome/ 10. (eating adj1 disorder*).kf,tw 11. (anorexi* or bulimi*).kf,tw 12. (diabulimi* or T1ED).kf,tw 13. ((binge or binging or bingeing or binge-eating or purge or purging) adj3 disorder*).kf,tw 14. pica.kf,tw 15. ((avoidant or restrictive) adj1 food intake disorder*).kf,tw 16. ARFID.kf,tw 17. ((rumination or regurgitation or rumination-regurgitation) adj1 syndrome).kf,tw 18. merycism.kf,tw 19. night eating syndrome.kf,tw 20. 1 or 2 or 3 or 4 or 5 or 6 or 7 or 8 21. 9 or 10 or 11 or 12 or 13 or 14 or 15 or 16 or 17 or 18 or 19 22. 20 and 21
*Embase (Ovid)*
1. work/ or job performance/ 2. return to work/ 3. occupation/ or career/ or employment/ or vocation/ or telecommuting/ or employment status/ 4. absenteeism/ or burnout/ or presenteeism/ or productivity/ or workload/ 5. work capacity/ or personnel management/ or employability/ 6. work disability/ 7. sheehan disability scale/ or "social and occupational functioning assessment scale"/ 8. (work* or job* or employ* or career* or occupation* or vocation* or profession* or telework* or telecommut*).ab,kw,ti 9. (absenteeism or presenteeism or (sick* adj2 presen*) or burnout).ab,kw,ti 10. ((capacity or disability or function*) adj2 (evaluation or assessment or scale)).ab,kw,ti 11. eating disorder/ or anorexia nervosa/ or avoidant restrictive food intake disorder/ or binge eating disorder/ or bulimia/ or pica/ or purging disorder/ 12. (eating adj1 disorder*).ab,kw,ti 13. (anorexi* or bulimi*).ab,kw,ti 14. (diabulimi* or T1ED).ab,kw,ti 15. ((binge or binging or bingeing or binge-eating or purge or purging) adj3 disorder*).ab,kw,ti 16. pica.ab,kw,ti 17. ((avoidant or restrictive) adj1 food intake disorder*).ab,kw,ti 18. ARFID.ab,kw,ti 19. ((rumination or regurgitation or rumination-regurgitation) adj1 syndrome).ab,kw,ti 20. merycism.ab,kw,ti 21. night eating syndrome.ab,kw,ti 22. 1 or 2 or 3 or 4 or 5 or 6 or 7 or 8 or 9 or 10 23. 11 or 12 or 13 or 14 or 15 or 16 or 17 or 18 or 19 or 20 or 21 24. 22 and 23
*CINAHL (Ebsco)*
1. (MH "Work") OR (MH "Job Performance") 2. MH "Job Re-Entry" 3. (MH "Occupations and Professions") OR (MH “Employment Status”) OR (MH "Employment of Disabled") OR (MH "Employment, Supported") OR (MH "Employment") OR (MH "Employment of Women") OR (MH "Part Time Employment") OR (MH "Self Employment") OR (MH "Temporary Employment") 4. (MH "Absenteeism") OR (MH "Presenteeism") OR (MH "Productivity") OR (MH "Burnout, Professional") OR (MH "Quality of Working Life") OR (MH “Workload”) 5. (MH "Employee Performance Appraisal+") OR (MH "Work Capacity Evaluation") 6. TI ( work* or job* or employ* or career* or occupation* or vocation* or profession* or telework* or telecommut*) OR AB ( work* or job* or employ* or career* or occupation* or vocation* or profession* or telework* or telecommut*) 7. TI ( absenteeism or presenteeism or (sickness N2 presen*) or burnout) OR AB ( absenteeism or presenteeism or (sickness N2 presen*) or burnout) 8. TI ( (capacity or disability or function*) N2 (evaluation or assessment or scale)) OR AB ( (capacity or disability or function*) N2 (evaluation or assessment or scale)) 9. (MH "Eating Disorders") OR (MH "Anorexia") OR (MH "Anorexia Nervosa") OR (MH "Binge Eating Disorder") OR (MH "Avoidant Restrictive Food Intake Disorder") OR (MH "Bulimia") OR (MH "Bulimia Nervosa") OR (MH "Night Eating Syndrome") OR (MH "Pica") 10. TI ( eating N1 disorder*) OR AB ( eating N1 disorder*) 11. TI ( (anorexi* or bulimi*)) OR AB ( (anorexi* or bulimi*)) 12. TI ( (binge or binging or bingeing or binge-eating or purge or purging) N3 disorder*) OR AB ( (binge or binging or bingeing or binge-eating or purge or purging) N3 disorder*) 13. TI pica OR AB pica 14. TI ( (avoidant or restrictive) N1 food intake disorder*) OR AB ( (avoidant or restrictive) N1 food intake disorder*) 15. TI ARFID OR AB ARFID 16. TI ( (rumination or regurgitation or rumination-regurgitation) N1 syndrome) OR AB ( (rumination or regurgitation or rumination-regurgitation) N1 syndrome) 17. TI ( diabulimi* or T1ED or “night eating syndrome”) OR AB ( diabulimi* or T1ED or “night eating syndrome”) 18. 1 or 2 or 3 or 4 or 5 or 6 or 7 or 8 19. 9 or 10 or 11 or 12 or 13 or 14 or 15 or 16 or 17 20. 18 and 19
*Web of Science*
1. TI=(work* or job* or employ* or career* or occupation* or vocation*or profession* or telework* or telecommut*) 2. TI=(absenteeism or presenteeism or (sick* near/2 presen*) or burnout) 3. TI=((capacity or disability or function*) near/2 (evaluation or assessment or scale)) 4. AB=(work* or job* or employ* or career* or occupation* or vocation*or profession* or telework* or telecommut*) 5. AB=(absenteeism or presenteeism or (sick* near/2 presen*) or burnout) 6. AB=((capacity or disability or function*) near/2 (evaluation or assessment or scale)) 7. AK=(work* or job* or employ* or career* or occupation* or vocation*or profession* or telework* or telecommut*) 8. AK=(absenteeism or presenteeism or (sick* near/2 presen*) or burnout) 9. AK=((capacity or disability or function*) near/2 (evaluation or assessment or scale)) 10. KP=(work* or job* or employ* or career* or occupation* or vocation*or profession* or telework* or telecommut*) 11. KP=(absenteeism or presenteeism or (sick* near/2 presen*) or burnout) 12. KP=((capacity or disability or function*) near/2 (evaluation or assessment or scale)) 13. TI=(eating near/1 disorder*) 14. TI=(anorexi* or bulimi* or diabulimi* or T1ED or pica or ARFID or merycism or “night eating syndrome”) 15. TI=((binge or binging or bingeing or binge-eating or purge or purging) near/3 disorder*) 16. TI=((avoidant or restrictive) near/1 “food intake disorder*”) 17. TI=((rumination or regurgitation or “rumination-regurgitation”) near/1 syndrome) 18. AB=(eating near/1 disorder*) 19. AB=(anorexi* or bulimi* or diabulimi* or T1ED or pica or ARFID or merycism or “night eating syndrome”) 20. AB=((binge or binging or bingeing or binge-eating or purge or purging) near/3 disorder*) 21. AB=((avoidant or restrictive) near/1 “food intake disorder*”) 22. AB=((rumination or regurgitation or “rumination-regurgitation”) near/1 syndrome) 23. AK=(eating near/1 disorder*) 24. AK=(anorexi* or bulimi* or diabulimi* or T1ED or pica or ARFID or merycism or “night eating syndrome”) 25. AK=((binge or binging or bingeing or binge-eating or purge or purging) near/3 disorder*) 26. AK=((avoidant or restrictive) near/1 “food intake disorder*”) 27. AK=((rumination or regurgitation or “rumination-regurgitation”) near/1 syndrome) 28. KP=(eating near/1 disorder*) 29. KP=(anorexi* or bulimi* or diabulimi* or T1ED or pica or ARFID or merycism or “night eating syndrome”) 30. KP=((binge or binging or bingeing or binge-eating or purge or purging) near/3 disorder*) 31. KP=((avoidant or restrictive) near/1 “food intake disorder*”) 32. KP=((rumination or regurgitation or “rumination-regurgitation”) near/1 syndrome) 33. 1 or 2 or 3 or 4 or 5 or 6 or 7 or 8 or 9 or 10 or 11 or 12 34. 13 or 14 or 15 or 16 or 17 or 18 or 19 or 20 or 21 or 22 or 23 or 24 or 25 or 26 or 27 or 28 or 29 or 30 or 31 or 32 35. 33 and 34
*APA PsychINFO (Ovid)*
1. exp job performance/ 2. reemployment/ 3. employment status/ or self-employment/ or supported employment/ or telecommuting/ or occupations/ 4. employee absenteeism/ or occupational stress/ or "quality of work life"/ or work load/ 5. vocational evaluation/ or employability/ 6. (work* or job* or employ* or career* or occupation* or vocation* or profession* or telework* or telecommut*).ti,ab,id 7. (absenteeism or presenteeism or (sick* adj2 presen*) or burnout).ti,ab,id 8. ((capacity or disability or function*) adj2 (evaluation or assessment or scale)).ti,ab,id 9. "feeding and eating disorders"/ or anorexia nervosa/ or avoidant restrictive food intake disorder/ or binge-eating disorder/ or bulimia nervosa/ or diabulimia/ or night eating syndrome/ or pica/ or rumination syndrome/ 10. (eating adj1 disorder*).ti,ab,id 11. (anorexi* or bulimi*).ti,ab,id 12. (diabulimi* or T1ED).ti,ab,id 13. ((binge or binging or bingeing or binge-eating or purge or purging) adj3 disorder*).ti,ab,id 14. pica.ti,ab,id 15. ((avoidant or restrictive) adj1 food intake disorder*).ti,ab,id 16. ARFID.ti,ab,id 17. ((rumination or regurgitation or rumination-regurgitation) adj1 syndrome).ti,ab,id 18. merycism.ti,ab,id 19. night eating syndrome.ti,ab,id 20. 1 or 2 or 3 or 4 or 5 or 6 or 7 or 8 21. 9 or 10 or 11 or 12 or 13 or 14 or 15 or 16 or 17 or 18 or 19 22. 20 and 21
*Cochrane Library (Wiley)*
1. [mh ^“work”] 2. [mh “work performance”] 3. [mh “return to work”] 4. [mh ^“employment”] 5. [mh “employment, supported”] 6. [mh “teleworking”] 7. [mh ^“occupations”] 8. [mh “absenteeism”] 9. [mh ^“efficiency”] 10. [mh “presenteeism”] 11. [mh “burnout, professional”] 12. [mh “workload”] 13. [mh “work capacity evaluation”] 14. [mh “employee performance appraisal”] 15. (work* or job* or employ* or career* or occupation* or vocation* or profession* or telework* or telecommut*):ti,ab,kw 16. (absenteeism or presenteeism or (sick* near/2 presen*) or burnout):ti,ab,kw 17. ((capacity or disability or function*) near/2 (evaluation* or assessment* or scale*)):ti,ab,kw 18. [mh ^"feeding and eating disorders"] 19. [mh “anorexia nervosa”] 20. [mh “avoidant restrictive food intake disorder”] 21. [mh “binge-eating disorder”] 22. [mh “bulimia nervosa”] 23. [mh “diabulimia”] 24. [mh “night eating syndrome”] 25. [mh “pica”] 26. [mh “rumination syndrome”] 27. (eating near/1 disorder*):ti,ab,kw 28. (anorexi* or bulimi*):ti,ab,kw 29. (diabulimi* or T1ED):ti,ab,kw 30. ((binge or binging or bingeing or binge-eating or purge or purging) near/3 disorder*):ti,ab,kw 31. pica:ti,ab,kw 32. ((avoidant or restrictive) near/1”food intake disorder*”):ti,ab,kw 33. ARFID:ti,ab,kw 34. ((rumination or regurgitation or rumination-regurgitation) near/1 syndrome):ti,ab,kw 35. merycism:ti,ab,kw 36. “night eating syndrome”:ti,ab,kw 37. 1 or 2 or 3 or 4 or 5 or 6 or 7 or 8 or 9 or 10 or 11 or 12 or 13 or 14 or 15 or 16 or 17 38. 18 or 19 or 20 or 21 or 22 or 23 or 24 or 25 or 26 or 27 or 28 or 29 or 30 or 31 or 32 or 33 or 34 or 35 or 36 39. 37 and 38

## Appendix 2

See Table 2.

**Table 2:** Priori scores for each critical appraisal tool

RoB 2 tool for RCTs	Low risk of bias: judgement of low risk for all domains Moderate risk of bias: judgement of some concerns in 1 or 2 domains High risk of bias: judgement of high risk in 1 or more domain OR judgement of some concerns for 3 or more domains
Newcastle Ottawa Scale for cohort studies	Low risk of bias: 8–9 points Moderate risk of bias: 6–7 points High risk of bias: 5 or less points
Newcastle Ottawa Scale for case–control studies	Low risk of bias: 8–9 points Moderate risk of bias: 6–7 points High risk of bias: 5 or less points
JBI checklist for cross-sectional studies	Low risk of bias: 7–8 questions met Moderate risk of bias: 4–6 questions met High risk of bias: 3 or less questions met
JBI checklist for case series	Low risk of bias: 8–10 questions met Moderate risk of bias: 5–7 questions met High risk of bias: 4 or less questions met

## Abbreviations

ED: Eating disorders
DSM-5: Diagnostic and Statistical Manual of Mental Disorders 5th Edition
ICD-11: International Classification of Diseases Revision 11
AN: Anorexia nervosa
NEDA: National Eating Disorder Association
BN: Bulimia nervosa
PRISMA-P: Preferred Reporting Items for Systematic Reviews and Meta-Analyses Protocols
PRISMA: Preferred Reporting Items for Systematic Reviews and Meta-Analyses
OT: Occupational therapist
RoB 2: Cochrane’s Risk of Bias 2 tool
JBI: Joanna Briggs Institute
GRADE: Grading of Recommendations, Assessment, Development and Evaluations
WFOT: World Federation of Occupational Therapists
